# Outcome Predictors in First-Ever Ischemic Stroke Patients: A Population-Based Study

**DOI:** 10.1155/2014/904647

**Published:** 2014-12-25

**Authors:** Giovanni Corso, Edo Bottacchi, Piera Tosi, Laura Caligiana, Chiara Lia, Massimo Veronese Morosini, Paola Dalmasso

**Affiliations:** ^1^Department of Neurology, Stroke Unit Ospedale Regionale, Viale Ginevra, No., 11100 Aosta, Italy; ^2^Statistics Department, Ospedale Regionale, Viale Ginevra 3, 11100 Aosta, Italy; ^3^Department of Scienze della Sanità Pubblica, Università degli Studi di Torino, Via Verdi 8, 10124 Torino, Italy

## Abstract

*Background*. There is scant population-based information regarding predictors of stroke severity and long-term mortality for first-ever ischemic strokes. The aims of this study were to determine the characteristics of patients who initially presented with first-ever ischemic stroke and to identify predictors of severity and long-term mortality. *Methods*. Data were collected from the population-based Cerebrovascular Aosta Registry. Between 2004 and 2008, 1057 patients with first-ever ischemic stroke were included. Variables analysed included comorbidities, sociodemographic factors, prior-to-stroke risk factors, therapy at admission and pathophysiologic and metabolic factors. Multivariate logistic regression models, Kaplan-Meier estimates, and Cox proportional Hazards model were used to assess predictors. *Results*. Predictors of stroke severity at admission were very old age (odds ratio [OR] 2.98, 95% confidence interval [CI] 1.75–5.06), female gender (OR 1.73, 95% CI 1.21–2.40), atrial fibrillation (OR 2.76, 95% CI 1.72–4.44), low ejection fraction (OR 2.22, CI 95% 1.13–4.32), and cardioembolism (OR 2.0, 95% CI 1.36–2.93). Predictors of long-term mortality were very old age (hazard ratio [HR] 2.02, 95% CI 1.65–2.47), prestroke modified Rankin scale 3–5 (HR 1.82; 95% CI 1.46–2.26), Charlson Index ≥2 (HR 1.97; 95% CI 1.62–2.42), atrial fibrillation (HR 1.43, 95% CI 1.04–1.98), and stroke severity (HR 3.54, 95% CI 2.87–4.36). *Conclusions*. Very old age and cardiac embolism risk factors are the independent predictors of stroke severity. Moreover, these factors associated with other comorbid medical conditions influence independently long-term mortality after ischemic stroke.

## 1. Introduction

Ischemic stroke has many causes, clinical presentations, risk factors, courses, and outcomes [1]. Management and prognosis of patients with ischemic stroke is directly related to specific mechanisms of the ischemic stroke. In the acute phase of stroke, the most important predictors of outcome are stroke severity [2, 3] and patient age [4]. Severe strokes seem to be more frequently caused by cardiac emboli and less frequently by large-artery occlusive mechanisms [5]. Functional status prior to stroke onset, presence of comorbid medical conditions [2, 6–9], cognitive impairment, and reduced consciousness at onset may also predict a worse prognosis after stroke, although with weaker evidence. Few studies have systematically evaluated multimodal factors in unselected consecutive ischemic stroke patients. The aim of this study is to estimate the predictors of stroke severity in the acute phase of stroke and to identify the predictors of all-cause long-term mortality subsequent to a first-ever ischemic stroke within a population-based setting. To determine the predictors is of paramount importance for clinicians to identify patients who are at higher risk for more severe strokes and death.

## 2. Methods

The Cerebrovascular Aosta Registry (CARe) is a population-based registry recording first-ever strokes in all age groups for a geographically defined area, the Aosta Valley, Italy. The overall study design hasbeen published previously [10, 11]. We prospectively checked all cases for diagnosis from overlapping sources. No selection of patients was performed with regard to age, stroke severity, or comorbid medical conditions before admission. Patients with recurrent stroke, intracerebral or subarachnoid hemorrhage, subdural hematoma, or other causes mimicking stroke (trauma, infection or an intracranial malignant processes) were excluded. Hospital care is free, and a very high proportion (92.5%) of stroke patients were admitted to hospital.

The aims of this study were to determine the characteristics of patients who initially presented with ischemic stroke, and to identify predictors of stroke severity and long-term (all cause) mortality.

We retrieved medical history prior to the index stroke and the CHADS2 score [12] was calculated for all patients. The following variables were analysed: gender, age (categorized as <65, 65–74, 75–84, and ≥85), domestic arrangements (lives with others, alone, or in a community), clinical history, and medications. Vascular risk factors included history of hypertension, diabetes mellitus, ischemic heart disease (acute myocardial infarction, angina, or coronary revascularisation), low ejection fraction, transient ischemic attack (TIA), current or former smoking, hypercholesterolemia, and AF, either as a history of AF and/or AF diagnosed during the index admission with stroke by electrocardiography (ECG) and/or 24 hour ECG monitoring. At admission, patients were categorized as treated or nontreated with antithrombotic agents. Patients with AF were considered as treated when they were receiving the following treatments: antiplatelet drugs and had CHADS2 score from 0 to 1; warfarin therapy with INR 2.0 to 3.0 at the time of stroke and had CHADS2 ≥2. Comorbidity conditions in patients admitted with acute ischemic stroke were scored with the modified Charlson Index (CI) on the basis of hospital discharge ICD-9CM codes [13] and patient history obtained from standardized case report forms. A weight was assigned to each indicated diagnosis and each weight was added together to calculate the CI score. The score varied according to the severity of the disease: myocardial infarction, congestive heart failure, peripheral vascular disease, dementia, chronic pulmonary disease, connective tissue disease, ulcer disease, mild liver disease, and diabetes mellitus were weighted 1; diabetes with end-organ damage, moderate to severe renal disease, nonmetastatic solid tumor, leukaemia, lymphoma, and multiple myeloma were weighted 2; autoimmune deficiency syndrome and metastatic solid tumor were weighted 6. The CI score was dichotomized (low comorbidity ≤1 versus high ≥2). Prestroke disability was assessed by the modified Rankin scale (mRS) [14].

Stroke was defined according to the World Health Organisation (WHO) criteria [15]. Ischemic stroke was diagnosed with a combination of clinical criteria and brain imaging. All patients underwent brain CT scan without contrast upon admission to exclude intracerebral hemorrhage.

Follow-up MRI examination or brain CT scan was repeated 2 to 5 days after the index event. Stroke severity was evaluated in the acute phase of the initial stroke by a neurologist certified in the use of the National Institute of Health Stroke Scale (NIHSS). Stroke severity by NIHSS was categorized as mild (0–4), moderate (5–15), or severe (16–42). Stroke subtypes were defined using the Trial of ORG 10172 in Acute Stroke Treatment (TOAST) criteria [16]. Metabolic, hematologic parameters and vital signs were recorded in the registry on arrival and again at 24–48 h after stroke onset. The length of hospital stay was defined from the day of admission to a hospital ward to the day of discharge to either his/her own residence to nursing home or to another type of institution. Acute stroke management and secondary prevention in these patient followed current European Stroke Organization guidelines [17].

Discharged patients were followed up on an annual basis through neurological examination and review of records for further hospitalization. All cases were followed up; the last followup was performed in December 2012. Data on vital status were obtained from the Ufficio Anagrafe (UA). Mortality data were cross-checked with the list of stroke death ICD-9 codes registered by the Office of Legal Medicine. Case fatality within 28 days, 1 year, and 5 years was defined as the proportion of cases for which death occurred from stroke onset for all causes.

## 3. Data Analysis

Differences in patient characteristics, premorbid risk factors, and hospital investigations were assessed by *χ* ^2^ test (for categorical variables) or analysis of variance (for continuous variables).

The variables analysed were age, gender, body mass index (BMI), life conditions, prestroke dependency, comorbidities, NIHSS at admission, vascular risk factors, therapy prior to stroke, pathophysiologic and metabolic factors.

Multivariate logistic regression models were used to estimate the impact of possible determinants of stroke severity at admission. Differences between groups and effect of patient characteristics on clinical outcome were assessed using Chi-square test. Cox regression model was used to estimate the impact of possible determinants of survival.

Kaplan-Meier survival curves were generated to demonstrate the effect of determinants on long-term survival.

Statistical tests were considered significant when the *P* value was ≤ 0.05. Statistical analyses were performed using STATA software version 10.

## 4. Results

The present analysis focused only on patients with first-ever ischemic stroke, 1057 cases, registered between January 1, 2004, and December 31, 2008. Of these, 260 patients presented AF, of whom 235 (90%) were known to have AF and 25 (10%) were diagnosed with AF during admission. The mean age of all patients was 75.7 (SD, 12.7) years. The mean age in the AF group was 80.8 years (SD, 10.1), and 74 years (SD, 13.1) in the non-AF group (*P* < 0.001). At admission, only 27 (10.5%) patients with AF were adequately treated according to the current guidelines. Nineteen patients had contraindication to oral anticoagulants because of frequent falls, severe bleeding, and dementia.

Patient characteristics at time of initial stroke are presented in Table 1.

As far as stroke severity, multivariate logistic regression analysis identified very old age, female gender, AF, low ejection fraction (<35%), and cardioembolism as independent factors associated with severe stroke (NIHSS ≥ 16), Table 2.

The mean length of hospital stay for all patients was 15.8 (95% CI 14.8–16.8) days. Patients with AF had a mean length of stay of 20 days (95% CI 17.5–22.5) whereas patients without AF had a mean length of stay of 15.3 days (95% CI 14.2–16.4) (*P* < 0.001).

At discharge, oral anticoagulants were prescribed to 53% of the AF group, 12% of patients did not receive any antithrombotic treatment, whereas antiplatelet agents were given to 35% of the AF group as the only antithrombotic treatment and to 88% of the non-AF group (*P* < 0.0001).

The mean follow-up time was 3.3 years (95% CI 3.1–3.4). During this period, 513 (48.5%) deaths occurred, of which 74 during hospitalization (Table 3).

The hazard ratios for 28 day, 1 year, and 5 year mortality were 2.36 (95% CI 1.53–3.63, *P* < 0.001), 2.75 (95% CI 2.05–3.68, *P* < 0.001), and 2.35 (95% CI 1.90–2.93, *P* < 0.001), respectively.

As far as predictors of long-term mortality, the multivariate Cox regression analysis showed very old age, prestroke modified Rankin scale 3–5, Charlson Index ≥2, AF, and severe stroke as independent predictors, Table 4.

**Figure 1:**
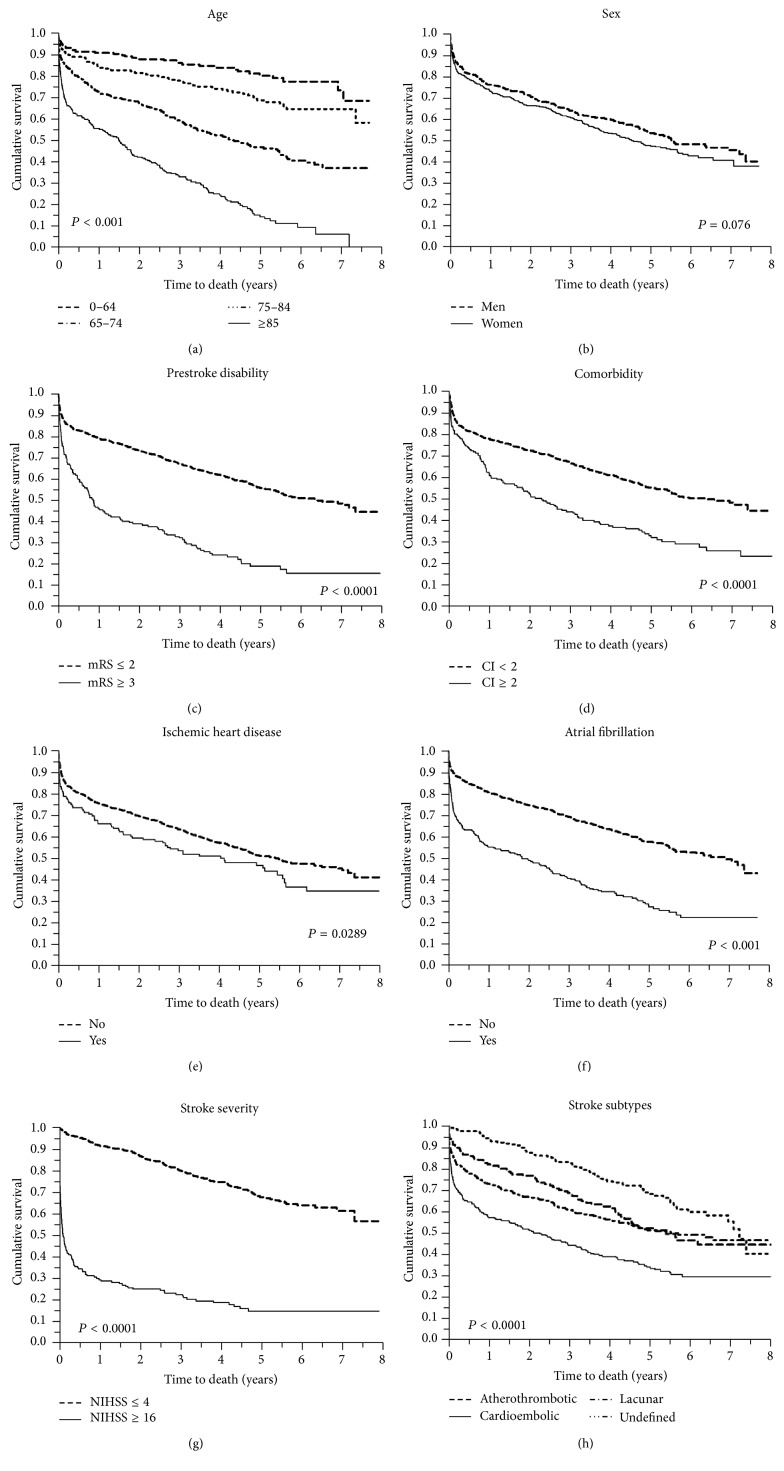
Kaplan-Meier survival curves indicating the relationship of (a) age, (b) sex, (c) prestroke disability (evaluated by mRS), (d) comorbidity (evaluated by Charlson Index, CI), (e) ischemic heart disease, (f) atrial fibrillation, (g) stroke severity, and (h) stroke subtypes with all-cause mortality after ischemic stroke.

Figure 1 shows the Kaplan-Meier curves after ischemic stroke representing the relationship of (a) age, (b) sex, (c) prestroke disability (evaluated by mRS), (d) comorbidity (evaluated by Charlson Index), (e) ischemic heart disease, (f) atrial fibrillation, (g) stroke severity, and (h) stroke subtypes with all-cause mortality after ischemic stroke.

## 5. Discussion

In this population-based study, on first-ever ischemic stroke patients, we demonstrated that risk factors such as very old age, AF, and cardioembolic stroke have an impact on neurologic impairment as evaluated in the acute phase and on long-term mortality. The majority of these factors are associated with high risk cardioembolic conditions. In the present study, very old age was found to be an independent predictor of stroke severity at admission. There have not been many community-based estimates of gender and older age as significant independent predictors of stroke severity, whereas very old age per se was found to be a strong predictor of outcome and mortality after stroke [18] and older age of patients with AF has been postulated as a major contributing factor for poor prognosis [5]. Female gender has been shown to have more severe strokes than men, as already reported in a previous review [19]. Conversely, our previous results showed that female patients were older and suffered more frequently from AF [11]; hence, female patients generally had a more severe stroke. We found cardioembolism to be the most common etiology of stroke (25.1%) in our almost exclusively white study population and cardioembolic stroke particularly dominated in the oldest age group. AF-related strokes are expected to increase due to population aging, because it is known the increase of AF with age [4, 6]. In our study, the mean age of patients with AF was older than in other studies [2, 20]. The well-documented impact of AF on the prognosis of first-ever ischemic stroke [2, 20–22] is confirmed in this investigation. Previous studies have reported that stroke patients with AF present more often large cortical infarcts, and less frequently lacunar infarction compared with patients without AF [2, 3, 5]. This may be explained by the size of cardiac emboli as well as by the lack of collateral vascularization, which may develop and compensate for acute arterial occlusion in patients with gradual occlusion of arteries, such as in atherosclerosis of cervical or cerebral arteries.

In this population study, most patients admitted with a stroke who had previously a diagnosis of AF were suboptimally anticoagulated before their stroke and only 10% of patients with a history of AF were receiving an adequate antithrombotic treatment prior to stroke. Other Italian studies on hospital admitted patients have reported underutilization of oral anticoagulant in patients with AF [23, 24]. Anticoagulation treatment is highly dependent on single patient characteristics such as age, comorbidities, patient's lifestyle, and feasibility of adequate monitoring of therapy [25]. The high percentage of nontreated patients is likely attributable to the older age of our population, being anticoagulation treatment difficult to control and to manage.

Several factors influenced long-term mortality among which prestroke dependency and comorbidities, more common in the elderly population. Prestroke mRS score 3–5 and a CI score >2 were found to be statistically significant independent factors of long-term mortality among first-ever stroke ischemic patients.

Many of the identified predictors of long-term mortality have been already reported in previous studies [3, 5, 18, 26–30] but hypertension, diabetes, or smoking. Hyperglycemia at the time of the index stroke was not associated with a worse outcome.

The strength of the present study is due to a community-based design and due to the use of rigorous case ascertainment procedures to enroll all patients with first-ever ischemic stroke. Complete case ascertainment allowed precise estimation of the prevalence of the risk factors among our patients. However, the limitations of our study must also be recognized: the Aosta Valley population is predominantly Caucasian, which may limit the possibility to generalize our findings to other ethnic groups, in whom the risk of AF and other risk factors may differ; the use of prestroke mRS is not standardized, because the mRS was designed and validated to measure global clinical function after stroke; our study did not examine the role and impact of hospital treatment practices, given the observational nature of this investigation.

In conclusion, the findings of this study suggest that very old age was a major contributing factor for poor prognosis in ischemic stroke patients. Presence of AF and cardioembolism were associated with severe stroke and reduced long-term survival. Moreover, long-term mortality after ischemic stroke may be accounted for the presence of prestroke dependency, comorbidity, or at least a history of ischemic heart disease.

## Figures and Tables

**Table 1:** Selected characteristics in the acute phase of 1057 patients with first-ever ischemic stroke.

	NIH 0–4	NIH 5–15	NIH ≥16	Total	*P*°
Number of patients	426	469	162	1057	
Gender female	199 (46.7%)	240 (51.2%)	105 (64.8%)	544 (51.5%)	0.0001^*^
Mean body mass index	25.5 (25.4-25.5)	25.22 (25.1-25.2)	24.3 (24.2-24.3)	25.2 (24.9–25.5)	0.0004^*^
Median age (interquartile range)	74.12 (64.6–80.4)	79.3 (72.3–85.3)	83.5 (74.2–89.9)	77.5 (69–84.4)	<0.0001^*^
Life conditions					
Lives with other (family)	330 (77.4%)	320 (68.2%)	105 (64.8%)	755 (71.4%)	0.002^*^
Lives alone	86 (71.4%)	118 (25.1%)	38 (8.1%)	242 (22.9%)	0.38
Lives in community	7 (5.7%)	27 (5.7%)	16 (3.4%)	50 (4.7%)	<0.0001^*^
Missing	3 (2.5%)	4 (1%)	3 (0.7%)	10 (1%)	
Vascular risk factors					
Arterial hypertension	322 (75.6%)	362 (77.2%)	118 (72.8%)	802 (75.9%)	0.49
Diabetes mellitus	74 (33.1%)	103 (21.9%)	23 (14.2%)	200 (18.9%)	0.35
Hypercholesterolemia	170 (39.9%)	147 (31.3%)	34 (21%)	351 (33.2%)	<0.0001^*^
Previous TIA	25 (5.8%)	27 (5.7%)	9 (5.5%)	61 (5.8%)	0.88
Current or former smoker	89 (20.9%)	59 (12.6%)	19 (11.7%)	167 (15.8%)	0.011^*^
Ischaemic heart disease	50 (11.7%)	63 (13.4%)	21 (13%)	134 (12.7%)	0.68
Atrial fibrillation	56 (13.1%)	127 (27.1%)	77 (47.5%)	260 (24.6%)	<0.0001^*^
Low ejection fraction (<35%)	21 (4.9%)	45 (9.6%)	20 (12.3%)	86 (8.1%)	0.002^*^
CHADS2 score					
0-1	68 (16%)	35 (7.5%)	20 (12.4%)	123 (11.6%)	0.3
≥2	358 (84%)	434 (92.5%)	142 (87.6%)	934 (88.4%)	0.2
Charlson Index score					
0-1	336 (78.8%)	345 (73.5%)	128 (79%)	809 (76.5%)	0.9
≥2	58 (13.6%)	98 (20.9%)	24 (14.8%)	180 (17%)	0.7
Missing	32 (7.6%)	26 (5.6%)	10 (6.2%)	68 (6.5%)	0.57
Prestroke disability (measured with mRS)					
0–2	404 (94.8%)	369 (78.7%)	117 (72.3%)	890 (84.2%)	<0.0001
3–5	18 (4.2%)	93 (19.8%)	42 (25.9%)	153 (14.6%)	<0.0001^*^
Missing	4 (1%)	7 (1.5%)	3 (1.8%)	14 (1.2%)	0.36
Home therapy					
Treated with antithrombotic agents	123 (28.8%)	135 (28.8%)	49 (30.2%)	307 (29%)	0.74
Nontreated with antithrombotic agents	303 (71.2%)	334 (71.2%)	113 (69.7%)	750 (71%)	0.74
Statins	38 (8.9%)	20 (4.3%)	4 (2.5%)	62 (5.9%)	0.76
Antihypertensive	242 (56.8%)	258 (55%)	85 (52.5%)	585 (55.4%)	0.34
Stroke onset during sleep	71 (16.6%)	109 (23.2%)	39 (24.1%)	219 (20.7%)	0.04
Metabolic values at admission					
Glucose (mg/dL)	114.2 (40.1)	125 (54.7)	126.4 (50.4)	120.9 (48.9)	0.0012^*^
LDL cholesterol (mg/dL)	117.3 (37.6)	112.6 (36.2)	102.1 (41.8)	113.4 (37.7)	<0.0001^*^
Systolic blood pressure (mmHg)	152.4 (24.3)	150.3 (24.1)	149.6 (24.6)	151 (24.5)	0.11
Diastolic blood pressure (mmHg)	87.3 (12.1)	86 (11.6)	85.4 (12.4)	86.43 (11.9)	0.044
TOAST classification of subtypes					
Large-artery atherosclerosis	66 (15.5%)	73 (15.5%)	14 (8.6%)	153 (14.5%)	0.032^*^
Cardioembolism	65 (15.2%)	122 (26%)	79 (48.8%)	266 (25%)	<0.0001^*^
Small-vessel occlusion lacun	109 (25.6%)	97 (20.7%)	4 (2.5%)	210 (20%)	<0.0001^*^
Stroke of undetermined etiology	175 (41.1%)	173 (36.9%)	64 (39.5%)	412 (39%)	0.7

Data are shown as number of patients (%) or mean (SD); TIA: transient ischaemic attack; mRS: modified Rankin scale; NIHSS: National Institute of Health Stroke Scale. CHADS2: Congestive heart failure, hypertension, age, diabetes mellitus, TIA, or thromboembolism. *P*° statistical analysis was performed on the NIHSS 0–4 and the NIHSS ≥16 groups. ^*^Variables included in the multivariate logistic regression analysis.

**Table 2:** Results of multivariate logistic regression analysis models for probability of NIHSS ≥16 score.

Variable	Odds ratio	95% CI	*P*
Sex female	1.73	1.21–2.40	0.0025
Age ≥85	2.98	1.75–5.06	0.0001
AF	2.76	1.72–4.44	<0.0001
Low ejection fraction (<35%)	2.22	1.13–4.32	0.0129
Cardioembolism	2.00	1.36–2.93	0.0004

CI: confidence interval; NIHSS: National Institutes of Health Stroke Scale; AF: atrial fibrillation.

**Table 3:** Mortality according to selected variables.

Variables		Mortality at 28 days	Mortality at 1 year	Mortality at 5 years	Alive	*P*
Gender	Male	48 (9.3%)	145 (28.3%)	236 (46%)	277 (54%)	
	Female	66 (12.1%)	122 (22.4%)	277 (50.9%)	267 (49.1%)	0.076

Age (year)	<65	7 (4.4%)	16 (10%)	35 (22%)	125 (78%)
	65–74	17 (6.4%)	39 (14.5%)	77 (28.8%)	191 (71.2%)
	75–84	44 (11.1%)	108 (27.3%)	209 (53%)	186 (47%)
	≥85	46 (19.6%)	104 (44.4%)	192 (81.6%)	42 (18.4%)	<0.0001
	Total	**114** (**10.8%**)	**267** (**25.2%**)	**513** (**48.5%**)	**544** (**51.5%**)	

Stroke severity (NIHSS)	0–4	4 (0.9%)	35 (8.2%)	133 (31.2%)	293 (68.8%)
	5–15	41 (8.7%)	118 (25.1%)	245 (52.2%)	224 (47.8%)
	≥16	69 (42.6%)	114 (70.4%)	135 (83.3%)	27 (16.7%)	<0.0001

Risk factor	AF yes	48 (18.5%)	115 (44.2%)	183 (70.4%)	77 (29.6%)	<0.0001
	AF no	66 (8.3%)	152 (19.1%)	330 (41.4%)	467 (58.6%)	

mRS before stroke	0-1	84 (9.6%)	196 (22.3%)	388 (44.2%)	489 (55.8%)	
	≥2	30 (16.6%)	71 (39.4%)	125 (69.4%)	55 (30.6%)	<0.0001

Charlson Index	0-1	84 (9.3%)	185 (20.4%)	392 (43.4%)	512 (56.6%)	
	≥2	30 (19.6%)	82 (53.6%)	121 (79.1%)	32 (20.9%)	<0.0001

Cardioembolism		50 (18.8%)	113 (42.5%)	175 (65.8%)	91 (34.2%)	<0.0001

Large-artery atherosclerosis		10 (6.5%)	28 (18.3%)	71 (46.4%)	82 (53.6%)	

Small-vessel occlusion		2 (0.9%)	13 (6.2%)	73 (34.2%)	137 (65.8%)	

Stroke of undetermined etiology		51 (12.4%)	112 (27.2%)	193 (46.8%)	219 (53.2%)	

Data are shown as number of patients (%); mRS: modified Rankin scale; NIHSS: National Institute of Health Stroke Scale.

**Table 4:** Cox proportional hazards analysis.

	Dependent variable: mortality		
	HR	95% CI	*P*
Covariate			
Age ≥85	2.02	1.65–2.47	<0.0001
Rankin before stroke 3–5	1.82	1.46–2.26	<0.0001
Charlson index ≥2	1.97	1.61–2.42	<0.0001
AF	1.43	1.04–1.98	0.0302
NIHSS ≥16	3.54	2.87–4.36	<0.0001
Large-artery atherosclerosis	4.90	0.68–35.10	0.1144
Cardioembolism	5.39	0.74–39.10	0.0966
Small-vessel occlusion	3.87	0.54–27.76	0.1789
Stroke of undetermined etiology	5.91	0.83–41.89	0.0764

HR: hazard ratio; C;. confidence interval; NIHSS: National Institutes of Health Stroke Scale; AF: atrial fibrillation.
